# The impact of shelf tags with Nutri-Score on consumer purchases: a difference-in-difference analysis of a natural experiment in supermarkets of a major retailer in Belgium

**DOI:** 10.1186/s12966-021-01207-7

**Published:** 2021-11-18

**Authors:** Stefanie Vandevijvere, Nicolas Berger

**Affiliations:** Sciensano, Service Lifestyle & Chronic Diseases, J.Wytsmanstraat 14, 1050 Brussels, Belgium

**Keywords:** Front-of-pack labeling, Electronic shelf labeling, Nutri-score, Supermarkets, Belgium

## Abstract

**Background:**

Delhaize, a major Belgian retailer, started implementing electronic shelf labels (ESL) with Nutri-Score since May 2019. Nutri-Score rates the healthfulness of packaged foods with five colours/letters from red/E (least healthy) to green/A (most healthy). This study evaluated the impact of ESL on consumer purchases, overall, and by food category.

**Methods:**

For 43 intervention stores (implementing ESL in the period 27 May 2019–19 June 2019), a control store, from the same province and retailer-assigned cluster was matched. There were 14 unique control stores. By store, weekly non-food and food sales for 2018 and 2019 were received by Nutri-Score (A/B/C/D/E) and food category according to a retailer-assigned classification system. The primary outcomes were the proportion of food sales for Nutri-Score A,B,C,D,E. Difference-in-differences regression analysis was conducted to estimate the effect of the ESL intervention on proportion of overall food and food category sales for Nutri-Score A,B,C,D,E, using linear mixed models to account for clustering at store-level. We controlled for store characteristics (region, cluster, non-food sales) and week of the year. Analyses were weighted to re-balance discrepancy between the number of intervention and control stores. To account for multiple testing, a Bonferroni adjustment was applied.

**Results:**

Comparing pre- and post-intervention periods, difference-in-differences for the proportion of Nutri-Score B and C product sales were more favourable in intervention than control stores (0.11 ± 0.04%, *p* = 0.007 and − 0.06 ± 0.03%, *p* = 0.026 respectively), while difference-in-differences for the proportion of Nutri-Score D product sales were less favourable in intervention than control stores (0.12 ± 0.04%, *p* = 0.002). For 17/58 food categories (representing 29% of total food sales) a positive impact [increase in healthier (Nutri-Score A, B) and/or decrease in less healthy (Nutri-Score D, E) food sales], and for 16/58 categories (representing 24% of total food sales) a negative impact was found. Positive impacts were found for vegetable, fruit and dairy products and confectionery. Negative impacts were found for bread and bakery products.

**Conclusion:**

The impact of ESL on consumer purchases was mixed. Favourable difference-in-differences were found for Nutri-Score B and C products and unfavourable difference-in-differences for Nutri-Score D products. Shelf labeling on its own is unlikely to significantly influence consumer behaviours.

## Introduction

Front-of-pack nutrition (FOP) labelling has been repeatedly recommended by the World Health Organization (WHO) as one of a suite of measures needed to improve population diets [1, 2]. The policy objectives of FOP labelling are generally twofold: (i) to provide interpretive information to consumers to inform healthier food choices; and (ii) to encourage the food industry to reformulate their products towards healthier options.

Nutri-Score on food packages was first implemented in France in October 2017, and then approved for implementation by the Minister of Public Health in Belgium in August 2018 and has been officially implemented in Belgium since April 1st 2019. The implementation of Nutri-Score on food packages is voluntary. All five biggest food retailers and a few food manufacturers have since either started or committed to put Nutri-Score on the food packages for their own brand products. At the end of 2019 about 10% of food products on the Belgian market displayed Nutri-Score on the package, of which 75% displayed Nutri-Score A or B [3]. The Nutri-Score label is attributed according to the calculation of a single and overall score that takes into account for every 100 g or 100 mL of food product: the amount of nutrients that should be limited (energy, saturated fat, total sugar, sodium), and the amount of nutrients and foods that should be encouraged (fibers, proteins, fruits, vegetables, pulses, nuts, and rapeseed, walnut and olive oils). Nutri-Score rates the nutrient content of packaged foods with five colours/letters from red/E (least healthy) to green/A (most healthy) [4, 5].

A previous study among a convenience sample of 1007 Belgian consumers found that Nutri-Score was the most effective FOP to inform consumers about the overall nutritional quality of food products, compared to other existing government-endorsed FOP nutrition labels internationally [6]. Research on the impact of Nutri-Score on consumer purchases or diets has so far been experimental and evidence of real life impact is limited. In France, the impact of Nutri-Score on purchasing intentions has been investigated using a randomized controlled trial in a virtual web-based supermarket [7] as well as in an experimental physical supermarket environment reproducing a physical grocery shop [8]. In the web-based supermarket, the intervention simulated shopping situations with front-of-pack nutrition labels affixed on food products. Around 12,000 participants were randomly assigned to one of five exposure conditions: Guideline Daily Amounts, Multiple Traffic Lights (MTL), Nutri-Score, Green Tick, or control (no front-of-pack exposure). The Nutri-Score significantly led to the highest overall nutritional quality of the shopping basket, followed by MTL and Green Tick, compared with the control, for all socio-economic groups. The Nutri-Score was also the only FOP label that led to significantly lower amounts in lipids, saturated fatty acids, and sodium of the shopping basket [7]. In the experimental supermarket, about 900 participants were recruited and distributed across three conditions: 1) control situation; 2) Application of the Nutri-Score on all breakfast cereals, sweet biscuits and appetizers; and 3) introduction of the Nutri-Score accompanied by consumer information on use and understanding of the label. Significantly higher mean nutritional quality was found of sweet biscuits purchased in the intervention combining the label and education, but not for the other food categories [8]. In a large multinational European cohort, consuming foods with a higher Food Standards Agency nutrient profiling system (FSAm-NPS) score (indicating lower nutritional quality), which grades the nutritional quality of food products and is used to derive Nutri-Score, was associated with a higher mortality from all causes and from cancer, as well as circulatory, respiratory, and digestive systems diseases [9].

Delhaize is one of the three grocery retailers with the largest market share (12.4%) in Belgium according to Euromonitor market share data [10]. It is the first supermarket chain in Belgium as well as internationally which started rolling out electronic shelf labels (ESL) with Nutri-Score for all food products (including own brand and other products) in-store across stores since May 2019. Such a natural experiment allows to give insights into the potential impact of mandatory Nutri-Score FOP labelling on consumer purchases in Belgium, with the difference that the label is put on a shelf tag rather than on the product package (the latter is only voluntary) and that it is black rather than in colour. A recent systematic review found that point-of-sale interventions identifying healthy/unhealthy food options can lead to healthier customer purchasing behavior, particularly those delivered using shelf-labels or technology [11]. The review included three shelf labeling studies exploring two types of on-shelf nutrition guidance systems [Guiding Stars: food healthiness depicted through gold stars, and NuVal: overall nutrition score for a food product calculated on a scale of 1 (least healthy) to 100 (most healthy) dependent on the content of fat, saturated fat, sodium, protein, dietary fibre, and various vitamins and minerals]. All three studies concluded that these interventions led to improved overall healthiness of consumer purchasing [11].

The aim of this study was to evaluate the impact of ESL with Nutri-Score on consumer purchases, overall, as well as for different food categories, in supermarkets from a major Belgian retailer.

## Methods

### Study design

This study was a controlled pre-to-post intervention assessment, which evaluated the impact of a real world retailer-initiated intervention on consumer food purchases. The researchers had no role in the design of this natural experiment. For this study, randomisation was not possible, but a group of control stores could be used for the evaluation, since the intervention was not rolled out in all Delhaize stores at the same time. A simple before-after analysis would not be valid as Delhaize began to implement Nutri-Score on food packages for its own brand products during the last quarter of 2018. Other food manufacturers in Belgium also started implementing the Nutri-Score on the FOP in 2019. This means that the number of food products with Nutri-Score displayed on the FOP increased steadily over time, which implied the need of a controlled design in order to assess the effect of the ESL intervention.

### Description of the natural experiment

**Fig. 1:**
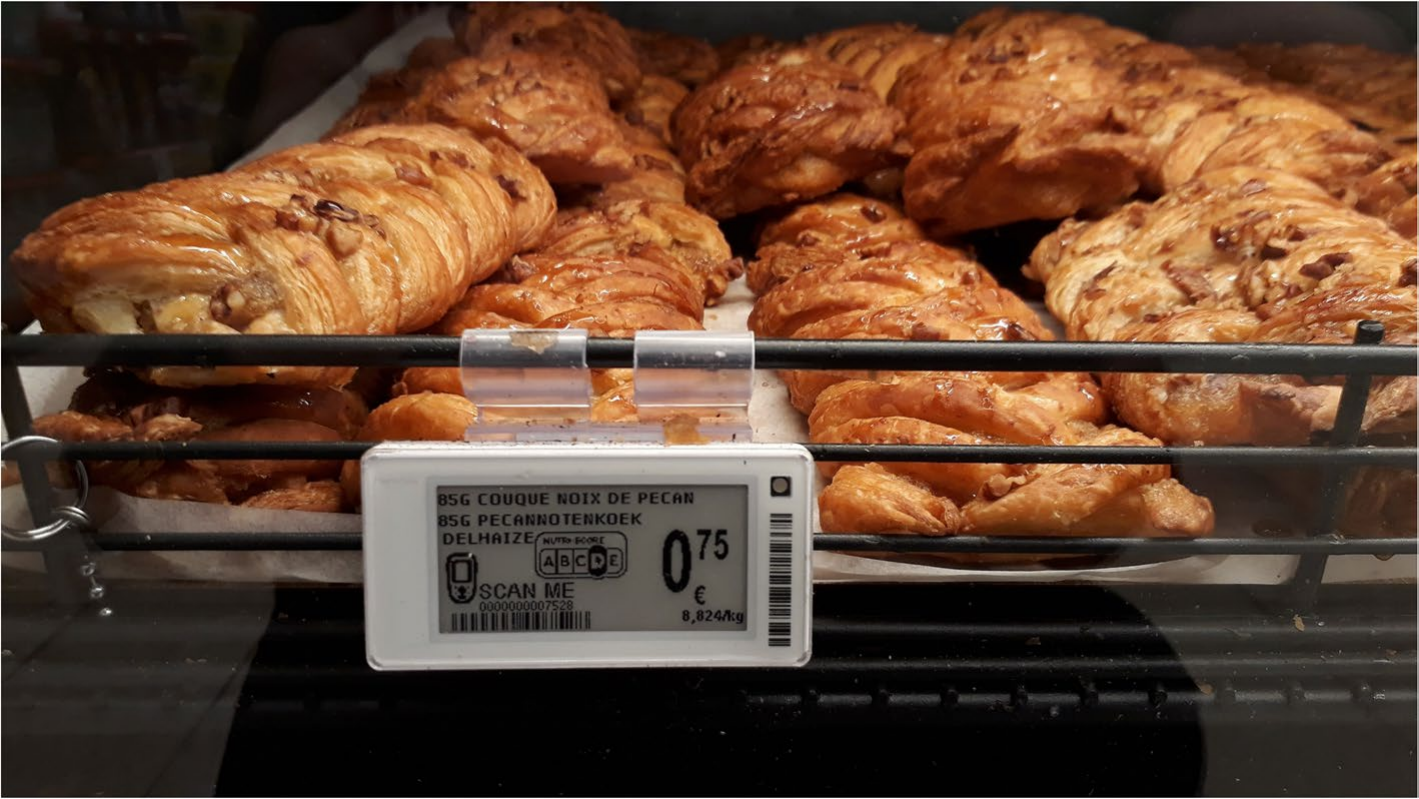
Black and white display of the Nutri-Score on electronic shelf labels (ESL) in-store

Since May 2019 Delhaize began rolling out electronic shelf labels (ESL) with Nutri-Score across its stores. Unlike the Nutri-Score on the food packages, the ESL are in black and white (Fig. 1). The ESL are included on the shelf tags for all food products for sale in-store, not only the retailers’ own brand products.

In addition to the ESL with Nutri-Score, Delhaize also ran national campaigns for Nutri-Score (in September 2018, January 2019 and September 2019), and offered price promotions in-store [20% reductions on all Delhaize products with Nutri-Score A/B for all loyalty card holders (September–December 2019) and 50% reductions on selected Delhaize products with Nutri-Score A/B, including fresh fruit and vegetables (May–August 2019)]. In this study the impact of the ESL was investigated over and above those other Nutri-Score related actions taken by the retailer.

### Selection of stores

There are in total 128 Delhaize stores across the country (65 in Flanders, 41 in Wallonia and 22 in Brussels). The retailer grouped stores in six different clusters based on census data, customer data and confidential store level data: Affluent Suburban, Cities (Flanders), Cities (Wallonia), Highly Urban, Highly Urban – small basket, Town/Village (Flanders). For 11 of the 128 stores, the introduction of an ESL with Nutri-Score was not possible over the period 2019–2020 due to older technology used. All other stores started with ESL within the period from 22 May 2019 to 22 October 2019, of which most stores (81%) started in June 2019 (Table 6 in Appendix 1).

The retailer confirmed that none of the stores had special issues (e.g. different portfolio of products, interventions on on-pack labelling and price promotions rolled out at different times/to a different extent to other stores, construction works) which were expected to affect the design of this study.

The retailer grouped all 128 stores by region, province and cluster (as explained above). For each store implementing the ESL in the period May–June 2019 (*N* = 112), it was evaluated whether a control store could be assigned from the same region, province and cluster, i.e. a store that either did not implement the ESL, or that implemented it at a later stage. In this way, pairs of intervention-control stores were formed. A control could be assigned to 43 of the 112 intervention stores. For some stores no control store could be assigned, because there was no store from the same region, province and cluster available with a later ESL implementation date, it was the only store available within the particular region, province and cluster or the implementation date of ESL was too close to implementation date of the ESL in the intervention store. Control stores were matched to more than one intervention store within the same region, province and cluster. In total there were 14 unique control stores with an ESL implementation date in September or October 2019 (*N* = 5) or with no ESL implementation (*N* = 9). For all 43 intervention stores, the ESL was implemented within a time frame of 3 weeks during May–June 2019 (27 May 2019–19 June 2019). The weekly sales for this period were excluded from the analysis.

### Food categories included

The food categories included in this study are given in Appendix 2. They are based on the food classification system used by the retailer. The food categories are ranked by average proportion of sales out of total food sales for both intervention as well as control stores (Appendix 2). Out of the total of 75 available food categories, the assessment as part of this study excluded herbs and spices, hot drinks, alcoholic drinks, diet products and meal replacements, specific groups of ethnic foods, gluten free products, as well as food categories with less than 3 out 5 different Nutri-Score categories, keeping 58 food categories in total.

### Data received from the retailer

For each store included in the study (43 intervention and 14 control stores), the weekly non-food and food sales data for the entire years 2018 and 2019 were obtained from the retailer by Nutri-Score allocation (A/B/C/D/E) and by food category according to the existing retailer food classification system (Appendix 2). The pre-intervention period was defined from 01 January 2018 to 20 May 2019 and the post-intervention period was defined from 27 June to 31 December 2019. According to the retailer any missing data for Nutri-Score on shelf labels were limited to less than 5% of the food and beverage products in scope for a Nutri-Score label. Since the same products are sold nationally in all Delhaize stores and since the data is centrally controlled, these missing data are similar for all stores at any time.

### Outcomes

The primary outcome measures assessed in this study were:

- % of total food sales for products with Nutri-Score A, B, C, D and E overall

Secondary outcome measures assessed in the study were:

- % of sales for food products with Nutri-Score A, B, C, D and E within food category
- % of sales for food products with Nutri-Score A, B, C, D and E by food category

### Data analysis

This study is a controlled pre-to-post intervention assessment. Difference-in-differences regression analysis was used to estimate the effect of the ESL intervention on proportion of food sales for Nutri-Score A,B,C,D,E. Such quasi-experimental method is increasingly used to evaluate the impact of interventions which could not be randomized [12]. We used linear mixed models to account for clustering at store-level over time. The model allowed for flexible time trends using binary variables for each week. We controlled for characteristics of the stores, including, region, cluster type, and total non-food sales (as a proxy for the size of the stores). Analyses were conducted for overall food sales, as well as by food category. In the model results, if the difference-in-difference coefficient estimate is statistically significant, it is likely the slopes in the control and intervention group are not parallel after the intervention, and so the exposure has affected the outcome in the exposed group differently than the underlying background trend, as captured by the unexposed group.

Analyses were weighted to re-balance the discrepancy between the number of intervention stores (*N* = 43) and controls (*N* = 14). Weights were assigned to each control store to reflect the frequency with which they were matched with a particular intervention store (x/43). The intervention stores all received the same weight (1/43).

In order to account for multiple testing, a Bonferroni adjustment was applied as the same analysis was repeated for multiple food categories. Therefore, the *p*-value was considered statistically significant if lower than 0.0008 (i.e. 0.05/58).

Significant substitutions (*p* < 0.0008) between Nutri-Score allocations (i.e. significant decrease in B and similar significant increase in A) within food categories were identified. The data were analyzed using SAS 9.4.

## Results

### Description of stores included in the study

The study included 43 intervention stores and 14 control stores. While weekly sales data for the entire years 2018 and 2019 were available for all included stores, for some stores the intervention period was shorter due to the implementation of ESL in the control store at a later date in 2019 (Table 1).

**Table 1:** Description of intervention and control stores included in the study, as well as the stores not included

		Intervention stores (*N* = 43)	Control stores (*N* = 14)	Stores not included (*N* = 71)
Region	Brussels	9	1	12
	Flanders	19	6	40
	Wallonia	15	7	19
Province	Antwerpen	3	1	16
	Brabant Wallon	1	1	6
	Brussels	9	1	12
	Hainaut	8	3	1
	Limburg	3	1	1
	Liège	2	1	11
	Luxembourg	1	1	1
	Namur	3	1	0
	Oost-Vlaanderen	0	0	13
	Vlaams-Brabant	6	2	7
	West-Vlaanderen	7	2	3
Cluster	Affluent Suburban	5	1	11
	Cities (Flanders)	8	3	11
	Cities (Wallonia)	13	6	12
	Highly Urban	0	0	18
	Highly Urban - Small basket	11	2	5
	Town/Village (Flanders)	6	2	14
ESL implementation	2019	43		
	*May 2019 (27–05/19–31/05/19)*	*3*		
	*June 2019 (01/06/19–19/06/19)*	*40*		
Intervention period taken into account (days)	Min	85		
	Max	218		
	Mean	173		
	Standard Deviation	47		

There were no baseline differences between intervention and control stores with regard to the proportion of food sales for different Nutri-Score categories, across the different seasons (Table 2).

**Table 2:** Baseline differences between control and intervention stores for average proportion (95%CI) of total weekly food sales for Nutri-Score A,B,C,D,E by season (year = 2018)

Control stores (*N* = 14)^a^		Intervention stores (*N* = 43)	
*Winter*		***Winter***	
*A*	33.3 (32.4–34.1)	*A*	34.1 (33.1–35.1)
*B*	19.8 (19.4–20.2)	*B*	19.6 (19.2–20.0)
*C*	14.3 (14.2–14.5)	*C*	14.1 (13.9–14.3)
*D*	26.3 (25.6–27.0)	*D*	27.6 (23.0–32.1)
*E*	15.0 (14.6–15.3)	*E*	14.4 (14.0–14.8)
*Spring*		***Spring***	
*A*	42.6 (41.7–43.5)	*A*	42.9 (41.6–44.1)
*B*	16.5 (16.2–16.9)	*B*	16.2 (15.8–16.6)
*C*	17.5 (17.2–17.8)	*C*	17.1 (16.7–17.5)
*D*	25.8 (25.2–26.3)	*D*	24.9 (24.3–25.5)
*E*	12.6 (12.2–12.9)	*E*	12.1 (11.8–12.5)
*Summer*		***Summer***	
*A*	40.0 (39.4–40.6)	*A*	40.9 (38.4–43.3)
*B*	17.1 (16.7–17.5)	*B*	16.9 (16.0–17.8)
*C*	17.7 (17.4–18.0)	*C*	17.8 (16.7–18.9)
*D*	25.5 (25.0–26.1)	*D*	25.2 (23.8–26.5)
*E*	12.8 (12.5–13.2)	*E*	12.5 (11.7–13.3)
*Autumn*		***Autumn***	
*A*	43.2 (42.4–44.1)	*A*	44.6 (43.6–45.7)
*B*	18.7 (18.2–19.2)	*B*	18.3 (17.9–18.7)
*C*	19.3 (19.0–19.6)	*C*	19.4 (19.0–19.7)
*D*	30.2 (29.4–30.9)	*D*	29.8 (29.1–30.4)
*E*	15.4 (15.1–15.8)	*E*	15.1 (14.7–15.5)

^a^Averages across stores taken, some stores get a higher weight as they are control store to multiple intervention stores

Seasons: winter (Jan/Feb/March), spring (April/May/June), summer (July/August/September) and autumn (October, November, December)

### Impact of ESL on proportion of total food sales for Nutri-score A, B, C, D, E overall, adjusted for region, cluster, week of the year and total non-food sales

Table 3 shows the overall results from the difference-in-difference analysis. There was no evidence of differences in the proportion of total food sales for Nutri-Score A, B, C, D and E between intervention and control stores prior to the intervention (Table 3).

**Table 3:** Impact of ESL on proportion of total weekly food sales for Nutri-Score A, B, C, D, E products, unadjusted and adjusted for region, cluster, week of the year and total non-food sales

	% (SE) of total food sales						
	control		intervention		p	diff-in-diff	
Nutri-Score	before	after	before	after	baseline diff^1^	coeff^2^ (SE)	p
	Unadjusted						
A	32.9 ± 0.5	32.3 ± 0.6	33.7 ± 0.3	33.0 ± 0.4	0.173	−0.05 ± 0.52	0.928
B	13.5 ± 0.1	13.8 ± 0.2	13.4 ± 0.1	13.7 ± 0.1	0.370	0.08 ± 0.21	0.718
C	13.9 ± 0.1	13.8 ± 0.1	13.8 ± 0.1	13.7 ± 0.1	0.834	−0.06 ± 0.21	0.776
D	20.8 ± 0.3	21.4 ± 0.3	20.4 ± 0.2	21.1 ± 0.2	0.258	0.09 ± 0.32	0.786
E	10.5 ± 0.2	10.6 ± 0.2	10.3 ± 0.1	10.5 ± 0.1	0.262	0.05 ± 0.16	0.773
	Adjusted for region, cluster and total non-food sales						
A	33.1 ± 0.4	32.8 ± 0.5	34.0 ± 0.3	33.8 ± 0.4	0.058	0.11 ± 0.52	0.832
B	13.4 ± 0.1	13.9 ± 0.2	13.5 ± 0.1	14.1 ± 0.2	0.884	0.16 ± 0.21	0.450
C	13.8 ± 0.1	13.8 ± 0.1	13.8 ± 0.1	13.8 ± 0.1	0.746	−0.03 ± 0.21	0.898
D	20.9 ± 0.1	21.6 ± 0.2	20.6 ± 0.1	21.5 ± 0.2	0.132	0.16 ± 0.31	0.615
E	10.3 ± 0.1	10.5 ± 0.1	10.3 ± 0.1	10.5 ± 0.1	0.597	0.07 ± 0.16	0.669
	Adjusted for region, cluster, total non-food sales and week of the year						
A	32.9 ± 0.5	33.4 ± 1.0	33.3 ± 0.4	33.7 ± 0.9	0.316	−0.09 ± 0.06	0.176
B	13.7 ± 0.2	13.1 ± 0.6	13.6 ± 0.1	13.1 ± 0.6	0.521	0.11 ± 0.04	**0.007**
C	13.7 ± 0.2	13.7 ± 0.4	13.8 ± 0.2	13.6 ± 0.4	0.856	−0.06 ± 0.03	**0.026**
D	21.0 ± 0.2	20.9 ± 0.5	20.8 ± 0.2	20.8 ± 0.5	0.144	0.12 ± 0.04	**0.002**
E	10.4 ± 0.2	10.3 ± 0.5	10.3 ± 0.2	10.3 ± 0.5	0.546	0.04 ± 0.03	0.265

^1^ *p*-value for mean difference in outcome between intervention and control groups prior to the intervention (existing baseline differences)

^2^ Difference in difference estimator. It shows whether the expected mean change in % total sales for Nutri-Score A,B,C,D,E from before to after was different between control and intervention stores

According to the unadjusted results, there were no significant impacts of the ESL intervention on the proportion of total food sales for Nutri-Score A, B, C, D or E, neither when the analyses were adjusted for region, cluster and total non-food sales.

When the analyses were also adjusted for week of the year, comparing pre- and post-intervention periods, difference-in-differences for the proportion of total sales for Nutri-Score B and C products were more favourable in the intervention stores than the control stores (0.11 ± 0.04%, *p* = 0.007 and − 0.06 ± 0.03%, *p* = 0.026 respectively). The decrease in proportion of sales for Nutri-Score B products from before to after was smaller in the intervention stores (from 13.6 ± 0.1% to 13.1 ± 0.6%) than the control stores (from 13.7 ± 0.2% to 13.1 ± 0.6%), while the decrease in proportion of sales for Nutri-Score C products was greater in the intervention stores (from 13.8 ± 0.2% to 13.6 ± 0.4%) than in the control stores (from 13.7 ± 0.2% to 13.7 ± 0.4%). Comparing pre- and post-intervention periods, difference-in-differences for the proportion of total sales for Nutri-Score D products were less favourable in the intervention stores than the control stores (0.12 ± 0.04%, *p* = 0.002). There was a decrease in proportion of sales for Nutri-Score D products from before to after in the control stores (from 21.0 ± 0.2% to 20.9 ± 0.5%) but not in the intervention stores (from 20.8 ± 0.2% to 20.8 ± 0.5%) (Table 3).

### Impact of ESL on proportion of sales of Nutri-score A, B, C, D, E products within food category, adjusted for region, cluster, week of the year and total non-food sales

The difference-in-difference coefficients for the impact of ESL on proportion of sales of Nutri-Score A, B, C, D, E products within food category, adjusted for region, cluster, week of the year and total non-food sales, by food category and Nutri-Score category are shown in Table 4. The unadjusted results can be found in Appendix 3.

**Table 4:**
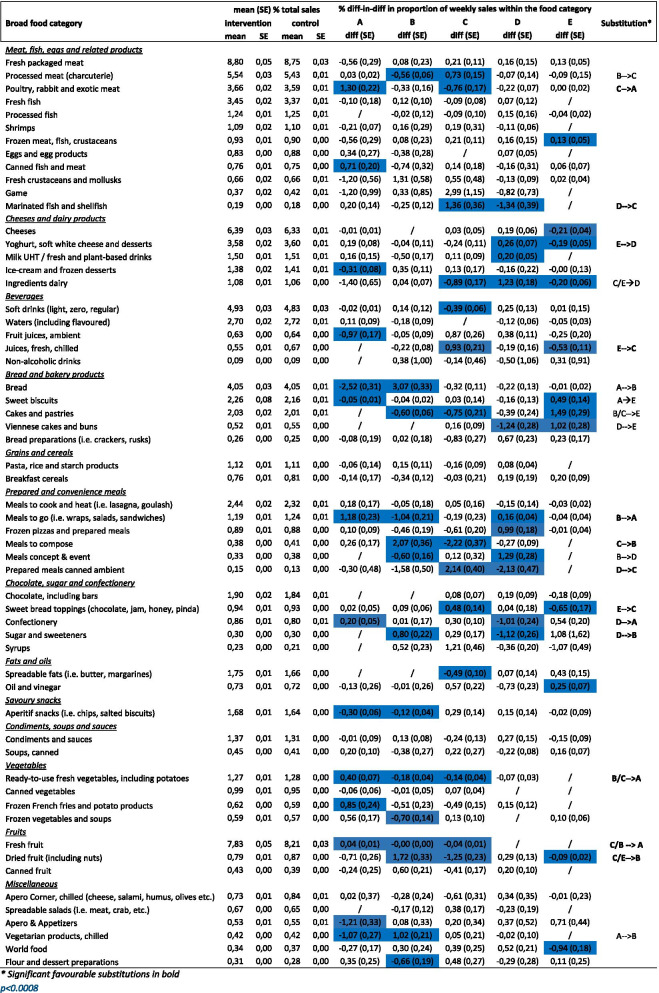
Impact of ESL on proportion of weekly sales for Nutri-Score A, B, C, D, E products within food category, by food category, adjusted for region, cluster, week and non-food sales

The mean change in the proportion of the food sales for Nutri-Score A within food category from before to after, adjusted for region, cluster, non-food sales and week of the year, was significantly higher (*p* < 0.0008) for intervention than control stores for poultry, rabbit and exotic meat (1.30 ± 0.22%), canned fish and meat (0.71 ± 0.20%), meals to go (i.e. wraps sandwiches) (1.18 ± 0.23%), confectionery (0.20 ± 0.05%), ready to use fresh vegetables and potatoes (0.40 ± 0.07%), frozen French fries and potato products (0.85 ± 0.24%), and fresh fruit (0.04 ± 0.01%). Similar mean changes were noted for other food categories (yoghurt, soft white cheese and desserts, canned soups, frozen vegetables and soups) but these weren’t statistically significant after the Bonferroni correction (*p* < 0.05 but > 0.0008) (Table 4).

The mean change in the proportion of the food sales for Nutri-Score B within food category from before to after, adjusted for covariates, was significantly higher (*p* < 0.0008) for intervention than control stores for bread (3.07 ± 0.33%), meals to compose (2.07 ± 0.36%), sugar and sweeteners (0.80 ± 0.22%), dried fruit including nuts (1.72 ± 0.33%) and chilled vegetarian products (1.02 ± 0.21%) (Table 4). Similar mean changes were noted for other food categories (fresh crustaceans and mollusks, ice-cream and frozen desserts, syrups, canned fruit) but these weren’t statistically significant after the Bonferroni correction (*p* < 0.05 but > 0.0008) (Table 4).

The mean change in the proportion of the food sales for Nutri-Score D within food category from before to after was significantly lower (*p* < 0.0008) for intervention than control stores for marinated fish and shell fish (− 1.34 ± 0.39%), Viennese cakes and buns (− 1.24 ± 0.28%), prepared meals canned ambient (− 2.13 ± 0.47%), confectionery (− 1.01 ± 0.24%), and sugar and sweeteners (− 1.12 ± 0.26%) (Table 4).

Similar mean changes were noted for other food categories (poultry rabbit and exotic meat, waters (including flavoured), meals to compose, oil and vinegar, canned soups, ready to use vegetables and potatoes) but these weren’t statistically significant after the Bonferroni correction (*p* < 0.05 but > 0.0008) (Table 4).

The mean change in the proportion of the food sales for Nutri-Score E within food category from before to after was significantly lower (*p* < 0.0008) for intervention than control stores for cheeses (− 0.21 ± 0.04%), yoghurt, soft white cheese and desserts (− 0.19 ± 0.05%), ingredients dairy (− 0.20 ± 0.06%),%), juices fresh chilled (− 0.53 ± 0.11%), sweet bread toppings (− 0.65 ± 0.17%), dried fruit (− 0.09 ± 0.02%) and world food (− 0.94 ± 0.18%) (Table 4). Similar mean changes were noted for other food categories (processed fish, diet products, meal replacements, special foods and syrups) but these weren’t statistically significant after the Bonferroni correction (*p* < 0.05 but > 0.0008) (Table 4).

Based on this analysis within food categories, favourable significant substitutions in proportion of sales towards healthier versions of food products were found for poultry, rabbit and exotic meat (Nutri-Score C to A), marinated fish and shell fish (Nutri-Score D to C), yoghurt, soft white cheeses and desserts (Nutri-Score E to D), juices fresh chilled (Nutri-Score E to C), Meals to go (i.e. wraps, salads, sandwiches) (Nutri-Score B to A), meals to compose (Nutri-Score C to B), prepared meals canned ambient (Nutri-Score D to C), Sweet bread toppings (chocolate, jam, honey, pinda) (Nutri-Score E to C), confectionery (Nutri-Score D to A), sugar and sweeteners (Nutri-Score D to B), Ready-to-use fresh vegetables, including potatoes (Nutri-Score B, C to A), fresh fruit (Nutri-Score B, C to A), and dried fruit including nuts (Nutri-Score C, E to B) (Table 4).

There were also some unfavourable significant substitutions for the food categories processed meat (Nutri-Score B to C), bread (Nutri-Score A to B), cakes and pastries (Nutri-Score B, C to E), Viennese cakes and buns (Nutri-Score D to E) and chilled vegetarian products (Nutri-Score A to B) (Table 4).

### Impact of ESL on proportion of total sales of Nutri-score A, B, C, D, E products by food category, adjusted for region, cluster, week of the year and total non-food sales

Table 5 shows the difference in difference coefficients adjusted for region, cluster, non-food sales and week of the year for the expected mean change in the proportion of total food sales for Nutri-Score A products, Nutri-Score B products, Nutri-Score C products, Nutri-Score D products and Nutri-Score E products by food category from before to after the ESL intervention.

**Table 5:**
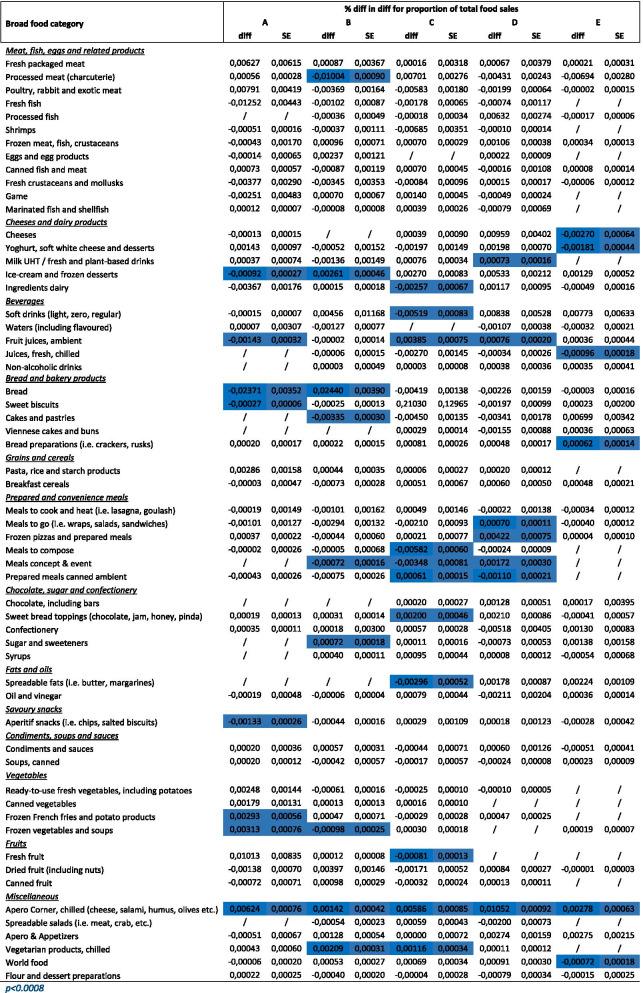
Difference-in-difference coefficients for proportion of total food sales for Nutri-Score A, B, C,D, E products by food category adjusted for region, cluster, total non-food sales and week of the year

The mean change in the proportion of total food sales for Nutri-Score A products from before to after was higher (*p* < 0.0008) for intervention than control stores for the following food categories: Frozen French fries and potato products, frozen vegetables and soups and Apero Corner chilled (cheese, salami, humus, olives etc.). The opposite was found for ice-cream and frozen desserts, ambient fruit juices, bread, sweet biscuits and Aperitif snacks (i.e. chips, salted biscuits) (Table 5).

The mean change in the proportion of total food sales for Nutri-Score B products from before to after was higher (*p* < 0.0008) for intervention than control stores for the following food categories: ice-cream and frozen desserts, bread, sugar and sweeteners, Apero Corner, chilled (cheese, salami, humus, olives etc.), and chilled vegetarian products. The opposite was found for: processed meat, hot drinks, cakes and pastries, and frozen vegetables and soups (Table 5).

The mean change in the proportion of total food sales for Nutri-Score D products from before to after was lower (*p* < 0.0008) for intervention than control stores for the following food categories: canned prepared ambient meals. The opposite was found for milk and plant based drinks, ambient fresh fruit juices, meals to go, frozen pizzas and prepared meals, and Apero Corner, chilled (cheese, salami, humus, olives etc.).

The mean change in the proportion of total food sales for Nutri-Score E products from before to after was lower (*p* < 0.0008) for intervention than control stores for the following food categories: cheeses, yoghurts soft white cheeses and desserts, juices fresh chilled, and world food. The opposite was found for: bread preparations, and Apero Corner, chilled (cheese, salami, humus, olives etc.) (Table 5).

## Discussion

This is the first study generating real world evidence of the impact of electronic shelf labels on all food products in-store on consumer food purchases. It was found that, overall, comparing pre- and post-intervention periods, difference-in-differences for the proportion of Nutri-Score B and C product sales were more favourable in intervention than control stores, while difference-in-differences for the proportion of Nutri-Score D product sales were less favourable in intervention than control stores. Across the 58 food categories investigated in the study, taking into account either significant changes in proportion of sales within food categories or significant changes in proportion of total sales for healthier (Nutri-Score A, B) and less healthy (Nutri-Score D, E) food products, no significant impact was found for 20/58 food categories, while for 17/58 food categories (representing about 29% of total food sales) a significant positive impact (either increase in healthier and/or decrease in less healthy food sales) was found. For 16/58 of food categories (representing 24% of total food sales) a significant negative impact (either decrease in healthier and/or increase in less healthy food sales) was found and for 5 food categories the impact was mixed. Positive impacts were found in particular for the groups of vegetables (i.e. fresh ready-to-use vegetables, frozen French fries and potato products and frozen vegetables and soups), fruits (fresh fruit, dried fruit including nuts), dairy products (cheeses, yoghurt soft white cheeses and desserts), and sugar and confectionery. Negative impacts were mainly found for the group of bread and bakery products.

Most previous studies used a simulated shopping situation to assess the impact of FOP or shelf labeling initiatives. A recent review reported that shoppers exposed to FOP or shelf labels had an increased intent to purchase healthier foods, such that FOP labels or shelf labels taken as a whole may achieve a small degree of success at persuading shoppers to buy healthier foods (< 2.0% shift in healthiness of food purchases) [13]. Studies carried out in real-world supermarkets, such as the present study, generate results that have more credibility than do studies in a simulated shopping situation as shoppers are not part of an experiment (which may influence their purchases) and they have more time to adapt to the intervention. Only a few studies have been conducted in real-world supermarkets. They used different types of shelf labeling schemes and found mixed results. Nutrient-specific systems like the traffic lights or sugar labels did not show an impact on purchases, while summary indicator systems like the Guiding Stars or NuVal did show an increase in sales of healthier foods. For example, a chain of supermarkets in the UK introduced Multiple Traffic Lights (MTL) on ready meals and sandwiches but revealed no shift to healthier products after 1 month’s time [14]. A similar study with MTL was carried out in an online supermarket in Australia [15], but there was no evidence that the FOP labels led to an increase in sales of healthier foods after 10 weeks. A recent study from the Netherlands evaluated the effectiveness of an industry-designed on-shelf sugar label on the sales of beverages with no, low, medium and high sugar content implemented within a real-world supermarket [16]. The study found that this label did not significantly decrease unhealthy beverage sales or significantly increase healthier beverage sales [16]. Two studies were carried out with Guiding stars attached to the shelves adjacent to the foods in supermarket chains in Canada and the USA. The Canadian study included a wide variety of foods [17]. Over the course of 6 months, relative to control supermarkets, shoppers in intervention supermarkets made small but significant shifts toward purchasing foods with higher nutritional ratings; however, shifts varied in direction and magnitude across food categories.

The other study was conducted in the USA and focused on the sales of ready-to-eat cereals [18]. Over the course of 7 months, sales of cereals with no stars fell by 2.6% (comparing stores carrying the logo with control stores) while sales of cereals with one, two, and three stars increased by 1.15, 0.89, and 0.54%, respectively. Another study in the US using the NuVAL shelf nutrition label, showed that after the change in the nutrient profile model underpinning the NuVAL (whereby average NuVAL scores decreased), yogurt sales declined. A 1-point increase in the NuVal score was estimated to increase sales of the corresponding yogurt product by 0.49% [19]. In the present study, the Nutri-Score, which is also a summary indicator system, was used as the shelf nutrition label. We found mixed results, dependent on the food categories and the Nutri-Score category studied. Our study is the first retailer-initiated natural experiment using Nutri-Score shelf labeling for all food products in-store. The impact of the ESL was investigated over and above other Nutri-Score related actions taken by the retailer. This could explain why the impact of the ESL intervention was not as consistent as that observed in previous studies using other summary indicator systems such as NuVal or Guiding Stars.

Shelf labeling interventions may not be able to overcome consumers’ strong preferences for particular foods and brands, and in-store displays and promotions may further reduce the effect of these interventions. Combining shelf labeling interventions with other nudges, such as price incentives or reductions in promotions for less healthy foods, are likely to be more effective [20]. It is important to note that the potential effect of the shelf labeling intervention, as measured in this study, may be lower than anticipated, due to the fact that other actions related to Nutri-Score (i.e. education and social marketing, voluntary front-of-pack labeling, price promotions on products with Nutri-Score A and B) were also in place over the course of the study.

While each of these particular actions could influence food purchases, they are unlikely to confound the effect of the ESL intervention because Delhaize displayed the Nutri-Score on-pack for new products in all stores at the same time and all other aforementioned Nutri-Score related actions took place at the same time across all Delhaize stores.

In addition, the ESL is in black and white, rather than in colour, such as with the Nutri-Score FOP. Previous studies found that Nutri-Score, with a summarized graded and color-coded format, using semantic colours, is associated to a higher objective understanding than monochrome and nutrient-specific labels [21]. The presence of colour in MTL and Nutri-Score labels is probably an additional reason for their effectiveness [22]. Although this has not been tested, the shelf labels, found on all products in store, could be an incentive for food companies to put Nutri-Score on their food packages and hence further stimulate reformulation efforts as well as increase impacts for the consumer.

There is no data on the potential population health impact of the intervention assessed in this study. A recent French study estimated that approximately 3.4% of all deaths from diet-related non-communicable diseases were avoidable due to implementation of the Nutri-Score FOP label on food packages in France [23].

Strengths of this study include the use of sales data for a comprehensive set of food groups and the use of a controlled design. Limitations include the reliance on retailer-defined food categories, the absence of available data on units and weights sold and the limited intervention period (longer period of follow-up not possible due to changes in purchase behaviour caused by the COVID-19 pandemic). Another limitation is that some intervention stores for which a control store could not be assigned were not included in the study.

There was no data available on in-store availability (for example the shelf-space allocation) for foods from each Nutri-Score category for the control and intervention stores. However, the retailer confirmed there were no interventions impacting availability of food products during the duration of the study and since we had matched control and intervention stores with respect to store characteristics the range of products available was likely similar.

In addition, even with a controlled design, it is possible that some other Nutri-Score related actions (i.e. FOP label, promotions, consumer social marketing) had differential effects in different shops, or with different populations, which this study may not have fully accounted for.

In conclusion, the impact of ESL with Nutri-Score on consumer purchases was mixed. Favourable difference-in-differences were found for Nutri-Score B and C products and unfavourable difference-in-differences for Nutri-Score D products. Shelf labeling on its own is unlikely to significantly influence consumer behaviours and should be supplemented with additional actions targeting marketing and prices.

## Data Availability

The sales data that support the findings of this study are available from Delhaize but restrictions apply to the availability of these data, which were used under license for the current study, and so are not publicly available. The data are however available from the authors upon reasonable request and with permission of Delhaize.

## Appendix 1

Table 6

**Table 6:** Implementation of Nutri-Score on ESL across the different Delhaize stores in Belgium

ESL implementation	N Stores
ESL not possible	11
May 2019	8
June 2019	104
September 2019	3
October 2019	2
Total	**128**

## Appendix 2

Table 7

**Table 7:** Food categories and their average % contributions to total food sales for control and intervention stores

Food category	Control stores		Intervention stores	
	Mean	stderr	mean	stderr
*Fresh packaged meat*	*8,799*	*0,050*	*8,746*	*0,027*
*Fresh fruit*	*7,834*	*0,046*	*8,205*	*0,028*
*Cheeses*	*6,393*	*0,026*	*6,328*	*0,013*
*Processed meat (charcuterie)*	*5,544*	*0,027*	*5,426*	*0,014*
*Soft drinks (light, zero, regular)*	*4,927*	*0,033*	*4,826*	*0,025*
*Bread*	*4,051*	*0,025*	*4,051*	*0,012*
*Poultry, rabbit and exotic meat*	*3,656*	*0,021*	*3,586*	*0,011*
*Yoghurt, soft white cheese and desserts*	*3,575*	*0,019*	*3,604*	*0,009*
*Fresh fish*	*3,451*	*0,022*	*3,374*	*0,012*
*Waters (including flavoured)*	*2,695*	*0,018*	*2,719*	*0,009*
*Meals to cook and heat (i.e. lasagna, goulash)*	*2,435*	*0,016*	*2,315*	*0,007*
*Sweet biscuits*	*2,263*	*0,079*	*2,161*	*0,006*
*Cakes and pastries*	*2,028*	*0,019*	*2,011*	*0,008*
*Chocolate, including bars*	*1,897*	*0,017*	*1,844*	*0,009*
*Spreadable fats (i.e. butter, margarines)*	*1,748*	*0,007*	*1,663*	*0,004*
*Aperitif snacks (i.e. chips, salted biscuits)*	*1,677*	*0,008*	*1,639*	*0,004*
*Milk UHT / fresh and plant-based drinks*	*1,495*	*0,011*	*1,512*	*0,006*
*Ice-cream and frozen desserts*	*1,375*	*0,019*	*1,409*	*0,011*
*Condiments and sauces*	*1,367*	*0,008*	*1,312*	*0,004*
*Ready-to-use fresh vegetables, including potatoes*	*1,268*	*0,007*	*1,276*	*0,003*
*Processed fish*	*1,237*	*0,012*	*1,252*	*0,006*
*Meals to go (i.e. wraps, salads, sandwiches)*	*1,193*	*0,014*	*1,242*	*0,007*
*Pasta, rice and starch products*	*1,121*	*0,007*	*1,114*	*0,004*
*Shrimps*	*1,093*	*0,016*	*1,098*	*0,009*
*Ingredients dairy*	*1,078*	*0,006*	*1,055*	*0,003*
*Canned vegetables*	*0,985*	*0,005*	*0,947*	*0,003*
*Sweet bread toppings (chocolate, jam, honey, pinda)*	*0,943*	*0,005*	*0,926*	*0,003*
*Frozen meat, fish, crustaceans*	*0,926*	*0,008*	*0,903*	*0,004*
*Frozen pizzas and prepared meals*	*0,886*	*0,007*	*0,876*	*0,003*
*Confectionery*	*0,857*	*0,006*	*0,799*	*0,006*
*Eggs and egg products*	*0,828*	*0,003*	*0,880*	*0,003*
*Dried fruit (including nuts)*	*0,786*	*0,006*	*0,869*	*0,004*
*Canned fish and meat*	*0,764*	*0,008*	*0,749*	*0,004*
*Breakfast cereals*	*0,764*	*0,005*	*0,814*	*0,003*
*Oil and vinegar*	*0,734*	*0,007*	*0,716*	*0,003*
*Apero Corner, chilled (cheese, salami, humus, olives etc.)*	*0,733*	*0,010*	*0,840*	*0,007*
*Spreadable salads (i.e. meat, crab, etc.)*	*0,671*	*0,004*	*0,651*	*0,002*
*Fresh crustaceans and mollusks*	*0,657*	*0,017*	*0,664*	*0,009*
*Fruit juices, ambient*	*0,627*	*0,003*	*0,641*	*0,002*
*Frozen French fries and potato products*	*0,617*	*0,004*	*0,585*	*0,002*
*Frozen vegetables and soups*	*0,591*	*0,005*	*0,569*	*0,002*
*Juices, fresh, chilled*	*0,553*	*0,005*	*0,668*	*0,004*
*Apero & Appetizers*	*0,525*	*0,013*	*0,552*	*0,007*
*Viennese cakes and buns*	*0,516*	*0,005*	*0,551*	*0,003*
*Soups, canned*	*0,448*	*0,004*	*0,408*	*0,002*
*Canned fruit*	*0,429*	*0,003*	*0,388*	*0,001*
*Vegetarian products, chilled*	*0,415*	*0,003*	*0,419*	*0,002*
*Meals to compose*	*0,382*	*0,004*	*0,410*	*0,002*
*Game*	*0,371*	*0,017*	*0,423*	*0,011*
*World food*	*0,340*	*0,003*	*0,370*	*0,002*
*Meals concept & event*	*0,330*	*0,003*	*0,380*	*0,003*
*Flour and dessert preparations*	*0,312*	*0,003*	*0,281*	*0,001*
*Sugar and sweeteners*	*0,303*	*0,002*	*0,296*	*0,001*
*Bread preparations (i.e. crackers, rusks)*	*0,259*	*0,002*	*0,245*	*0,001*
*Syrups*	*0,231*	*0,004*	*0,214*	*0,002*
*Marinated fish and shellfish*	*0,193*	*0,002*	*0,181*	*0,001*
*Prepared meals canned ambient*	*0,154*	*0,002*	*0,133*	*0,001*
*Non-alcoholic drinks*	*0,092*	*0,002*	*0,088*	*0,001*

## Appendix 3

Table 8

**Table 8:** Impact of ESL on proportion of weekly sales for Nutri-Score A, B, C, D, E products by food category, unadjusted and adjusted for region, cluster, week and non-food sales

Food category	Nutri-Score	Unadjusted			Adjusted for region, cluster, non-food sales			Adjusted for region, cluster, non-food sales, week of the year		
		*Estimate*	*StdErr*	*p*	*Estimate*	*StdErr*	*p*	*Estimate*	*StdErr*	*p*
Frozen meat, fish, crustaceans	A	-0.4751	0.5094	0.3511	-0.8723	0.4960	0.0787	-0.5587	0.2943	0.0577
Frozen meat, fish, crustaceans	B	0.0773	0.4464	0.8626	0.4705	0.4327	0.2769	0.0781	0.2331	0.7376
Frozen meat, fish, crustaceans	C	0.1950	0.1437	0.1749	0.2844	0.1415	0.0445	0.2054	0.1135	0.0705
Frozen meat, fish, crustaceans	D	0.0812	0.1869	0.6640	0.0625	0.1871	0.7383	0.1570	0.1479	0.2882
Frozen meat, fish, crustaceans	E	0.0802	0.0683	0.2405	0.0429	0.0674	0.5249	0.1253	0.0509	0.0138
Canned Fish and meat	A	0.7483	0.2358	0.0015	0.7485	0.2360	0.0015	0.7102	0.1954	0.0003
Canned Fish and meat	B	-0.6022	0.4096	0.1416	-0.5315	0.4095	0.1944	-0.7441	0.3175	0.0191
Canned Fish and meat	C	0.1958	0.2276	0.3897	0.2516	0.2275	0.2689	0.1398	0.1832	0.4454
Canned Fish and meat	D	-0.3492	0.4203	0.4062	-0.3922	0.4207	0.3513	-0.1611	0.3130	0.6067
Canned Fish and meat	E	0.0194	0.1037	0.8513	-0.0417	0.1021	0.6828	0.0560	0.0749	0.4545
Fresh packaged meat	A	0.2543	0.3044	0.4035	0.3969	0.3015	0.1881	0.4128	0.1699	0.0152
Fresh packaged meat	B	0.0168	0.2120	0.9367	-0.0185	0.2120	0.9305	-0.0113	0.1358	0.9335
Fresh packaged meat	C	0.0834	0.1596	0.6012	0.0719	0.1597	0.6526	0.0006	0.1292	0.9965
Fresh packaged meat	D	-0.3569	0.2688	0.1845	-0.4453	0.2678	0.0964	-0.4030	0.1543	0.0090
Fresh packaged meat	E	0.0025	0.0178	0.8880	-0.0076	0.0176	0.6661	-0.0000	0.0164	0.9988
Processed meat (charcuterie)	A	0.0089	0.0258	0.7307	0.0118	0.0259	0.6472	0.0279	0.0178	0.1164
Processed meat (charcuterie)	B	-0.5033	0.1261	<.0001	-0.5477	0.1256	<.0001	-0.5636	0.0574	<.0001
Processed meat (charcuterie)	C	0.6461	0.3227	0.0453	0.5539	0.3214	0.0848	0.7305	0.1483	<.0001
Processed meat (charcuterie)	D	-0.0706	0.2829	0.8029	0.1918	0.2730	0.4823	-0.0734	0.1425	0.6064
Processed meat (charcuterie)	E	-0.0617	0.2394	0.7966	-0.1657	0.2378	0.4860	-0.0907	0.1486	0.5416
Poultry, rabbit and exotic	A	1,1798	0.5248	0.0246	1,3083	0.5242	0.0126	1,3015	0.2245	<.0001
Poultry, rabbit and exotic	B	-0.2504	0.3559	0.4818	-0.3256	0.3556	0.3599	-0.3319	0.1586	0.0364
Poultry, rabbit and exotic	C	-0.6714	0.3639	0.0651	-0.6562	0.3642	0.0717	-0.7567	0.1658	<.0001
Poultry, rabbit and exotic	D	-0.2615	0.1060	0.0136	-0.3182	0.1047	0.0024	-0.2191	0.0671	0.0011
Poultry, rabbit and exotic	E	-0.0016	0.0230	0.9406	-0.0069	0.0219	0.7529	0.0005	0.0179	0.9772
Game	A	-0.7642	1,2069	0.5267	-1,1234	1,1990	0.3490	-1,2000	0.9991	0.2299
Game	B	0.0099	0.9684	0.9919	0.4846	0.9340	0.6040	0.3292	0.8514	0.6991
Game	C	3,1733	1,7959	0.0775	4,1104	1,7707	0.0204	2,9877	1,1456	0.0092
Game	D	-1,9164	2,1397	0.3707	-1,7999	2,1614	0.4052	-0.8218	0.7344	0.2634
Fresh fish	A	-0.1584	0.3281	0.6293	-0.0945	0.3279	0.7731	-0.1008	0.1820	0.5796
Fresh fish	B	0.1228	0.1390	0.3770	0.0244	0.1360	0.8574	0.1207	0.0981	0.2189
Fresh fish	C	-0.0978	0.1204	0.4166	-0.1660	0.1187	0.1621	-0.0911	0.0761	0.2316
Fresh fish	D	0.1275	0.2314	0.5816	0.2251	0.2295	0.3266	0.0695	0.1213	0.5667
Fresh crustaceans and mollusks	A	-1,4266	0.9833	0.1469	-1,3748	0.9847	0.1627	-1,2014	0.5575	0.0312
Fresh crustaceans and mollusks	B	1,8213	1,3615	0.1811	1,5804	1,3613	0.2457	1,3125	0.5751	0.0225
Fresh crustaceans and mollusks	C	0.5650	0.7529	0.4531	0.8894	0.7468	0.2338	0.5486	0.4794	0.2525
Fresh crustaceans and mollusks	D	-0.2009	0.1074	0.0616	-0.1687	0.1072	0.1156	-0.1341	0.0945	0.1559
Fresh crustaceans and mollusks	E	0.0592	0.0469	0.2072	0.0719	0.0467	0.1233	0.0241	0.0408	0.5541
Processed fish	B	0.0226	0.1465	0.8773	0.0769	0.1461	0.5986	-0.0209	0.1244	0.8667
Processed fish	C	-0.0367	0.1262	0.7714	0.0138	0.1256	0.9125	-0.0875	0.1017	0.3901
Processed fish	D	0.0349	0.2074	0.8665	-0.0542	0.2062	0.7928	0.1484	0.1633	0.3634
Processed fish	E	-0.0441	0.0222	0.0468	-0.0335	0.0218	0.1248	-0.0409	0.0208	0.0493
Shrimps	A	-0.2038	0.0825	0.0135	-0.1855	0.0825	0.0246	-0.2085	0.0689	0.0025
Shrimps	B	0.0128	0.4673	0.9782	0.0889	0.4675	0.8492	0.1553	0.2876	0.5893
Shrimps	C	0.2934	0.4998	0.5572	0.2182	0.5001	0.6626	0.1947	0.3108	0.5311
Shrimps	D	-0.1115	0.0635	0.0792	-0.0993	0.0635	0.1180	-0.1183	0.0608	0.0520
Marinated fish and shellfish	A	0.1905	0.1588	0.2305	0.3050	0.1551	0.0493	0.1908	0.1362	0.1611
Marinated fish and shellfish	B	-0.2788	0.1488	0.0611	-0.3025	0.1487	0.0419	-0.2517	0.1237	0.0419
Marinated fish and shellfish	C	1,4621	0.3835	0.0001	1,3944	0.3832	0.0003	1,3555	0.3608	0.0002
Marinated fish and shellfish	D	-1,4722	0.4257	0.0005	-1,4301	0.4257	0.0008	-1,3362	0.3868	0.0006
Eggs and egg products	A	0.2500	0.3701	0.4994	0.5426	0.3595	0.1313	0.3449	0.2744	0.2089
Eggs and egg products	B	-0.2920	0.3782	0.4402	-0.5978	0.3668	0.1032	-0.3813	0.2785	0.1710
Eggs and egg products	D	0.0544	0.0537	0.3114	0.0760	0.0535	0.1554	0.0659	0.0459	0.1510
Vegetarian products, chilled	A	-1,1428	0.2785	<.0001	-1,1136	0.2788	<.0001	-1,0669	0.2677	<.0001
Vegetarian products, chilled	B	1,0995	0.2315	<.0001	1,0056	0.2303	<.0001	1,0243	0.2120	<.0001
Vegetarian products, chilled	C	-0.0238	0.2387	0.9204	0.0481	0.2376	0.8394	0.0543	0.2126	0.7984
Vegetarian products, chilled	D	0.0527	0.1127	0.6401	0.0450	0.1128	0.6902	-0.0169	0.1046	0.8714
Milk UHT / fresh and plant-based drinks	A	0.2146	0.2365	0.3641	-0.0140	0.2260	0.9505	0.1690	0.1501	0.2601
Milk UHT / fresh and plant-based drinks	B	-0.5599	0.1969	0.0045	-0.6320	0.1961	0.0013	-0.5047	0.1689	0.0028
Milk UHT / fresh and plant-based drinks	C	0.1230	0.1998	0.5381	0.3897	0.1825	0.0328	0.1118	0.0939	0.2342
Milk UHT / fresh and plant-based drinks	D	0.1982	0.0542	0.0003	0.2298	0.0534	<.0001	0.2010	0.0459	<.0001
Yoghurt, soft white cheese and desserts	A	0.2846	0.1254	0.0233	0.2959	0.1255	0.0184	0.1853	0.0819	0.0236
Yoghurt, soft white cheese and desserts	B	-0.0456	0.1798	0.8000	0.0850	0.1760	0.6294	-0.0444	0.1079	0.6808
Yoghurt, soft white cheese and desserts	C	-0.3285	0.1905	0.0848	-0.3649	0.1904	0.0554	-0.2383	0.1049	0.0232
Yoghurt, soft white cheese and desserts	D	0.2027	0.1254	0.1061	0.1153	0.1221	0.3450	0.2632	0.0743	0.0004
Yoghurt, soft white cheese and desserts	E	-0.1632	0.0732	0.0258	-0.1629	0.0732	0.0262	-0.1884	0.0497	0.0002
Ingredients dairy	A	-1,5217	0.7654	0.0487	-1,5850	0.7654	0.0402	-1,4003	0.6491	0.0346
Ingredients dairy	B	-0.0121	0.0777	0.8762	-0.0160	0.0777	0.8371	0.0371	0.0672	0.5803
Ingredients dairy	C	-0.7586	0.2728	0.0054	-0.6417	0.2707	0.0178	-0.8870	0.1683	<.0001
Ingredients dairy	D	1,1407	0.2789	<.0001	1,0247	0.2767	0.0002	1,2328	0.1780	<.0001
Ingredients dairy	E	-0.1859	0.0608	0.0022	-0.1745	0.0608	0.0041	-0.2016	0.0581	0.0005
Cheeses	A	-0.0121	0.0090	0.1820	-0.0101	0.0091	0.2677	-0.0075	0.0088	0.3980
Cheeses	C	0.1762	0.1289	0.1718	0.2221	0.1282	0.0834	0.0260	0.0512	0.6112
Cheeses	D	0.0425	0.1280	0.7396	-0.0003	0.1273	0.9981	0.1874	0.0621	0.0026
Cheeses	E	-0.2177	0.0469	<.0001	-0.2193	0.0470	<.0001	-0.2138	0.0391	<.0001
Ice-cream and frozen desserts	A	-0.3340	0.1636	0.0412	-0.5417	0.1525	0.0004	-0.3092	0.0776	<.0001
Ice-cream and frozen desserts	B	0.4221	0.1756	0.0162	0.4583	0.1753	0.0090	0.3511	0.1091	0.0013
Ice-cream and frozen desserts	C	0.1622	0.2412	0.5014	0.2189	0.2412	0.3643	0.1323	0.1665	0.4269
Ice-cream and frozen desserts	D	-0.2685	0.3040	0.3771	-0.2351	0.3039	0.4391	-0.1637	0.2190	0.4547
Ice-cream and frozen desserts	E	0.0361	0.1725	0.8342	0.1163	0.1712	0.4967	-0.0027	0.1326	0.9838
Frozen French fries and potato products	A	1,4112	0.5543	0.0109	1,7677	0.5458	0.0012	0.8477	0.2415	0.0005
Frozen French fries and potato products	B	-0.7917	0.4046	0.0504	-1,1252	0.3935	0.0043	-0.5076	0.2289	0.0266
Frozen French fries and potato products	C	-0.6742	0.1944	0.0005	-0.6381	0.1944	0.0010	-0.4908	0.1489	0.0010
Frozen French fries and potato products	D	0.0593	0.1487	0.6900	0.0230	0.1483	0.8768	0.1488	0.1152	0.1965
Frozen Pizzas and prepared meals	A	0.0091	0.1199	0.9398	0.0337	0.1200	0.7791	0.1003	0.0918	0.2749
Frozen Pizzas and prepared meals	B	-0.3454	0.2728	0.2055	-0.3334	0.2731	0.2223	-0.4581	0.1918	0.0169
Frozen Pizzas and prepared meals	C	-0.4441	0.2499	0.0756	-0.3418	0.2477	0.1676	-0.6105	0.1960	0.0018
Frozen Pizzas and prepared meals	D	0.8190	0.3155	0.0095	0.6962	0.3140	0.0266	0.9901	0.1843	<.0001
Frozen Pizzas and prepared meals	E	-0.0108	0.0514	0.8335	-0.0192	0.0515	0.7098	-0.0130	0.0409	0.7507
Prepared meals canned ambient	A	-0.5470	0.4905	0.2648	-0.5033	0.4909	0.3053	-0.2953	0.4770	0.5360
Prepared meals canned ambient	B	-1,4361	0.5189	0.0057	-1,4405	0.5194	0.0056	-1,5831	0.5010	0.0016
Prepared meals canned ambient	C	2,3508	0.4102	<.0001	2,4037	0.4102	<.0001	2,1441	0.3956	<.0001
Prepared meals canned ambient	D	-2,3101	0.5062	<.0001	-2,3002	0.5061	<.0001	-2,1321	0.4673	<.0001
Aperitif snacks (i.e. chips, salted biscuits)	A	-0.2263	0.0763	0.0030	-0.2567	0.0758	0.0007	-0.3007	0.0582	<.0001
Aperitif snacks (i.e. chips, salted biscuits)	B	-0.1151	0.0392	0.0033	-0.1094	0.0392	0.0052	-0.1242	0.0368	0.0007
Aperitif snacks (i.e. chips, salted biscuits)	C	0.2406	0.2091	0.2498	0.2520	0.2092	0.2286	0.2870	0.1414	0.0424
Aperitif snacks (i.e. chips, salted biscuits)	D	0.1957	0.2103	0.3521	0.2527	0.2097	0.2282	0.1517	0.1436	0.2910
Aperitif snacks (i.e. chips, salted biscuits)	E	-0.1016	0.1220	0.4048	-0.1448	0.1214	0.2332	-0.0167	0.0869	0.8478
Apero, chilled (cheese, salami, humus, olives etc.)	A	0.2961	0.5323	0.5780	0.8249	0.5085	0.1048	0.0208	0.3742	0.9556
Apero, chilled (cheese, salami, humus, olives etc.)	B	-0.1694	0.3539	0.6323	0.1532	0.3347	0.6471	-0.2715	0.2439	0.2656
Apero, chilled (cheese, salami, humus, olives etc.)	C	-0.5789	0.4463	0.1946	-0.4948	0.4464	0.2677	-0.6093	0.3050	0.0458
Apero, chilled (cheese, salami, humus, olives etc.)	D	-0.0173	0.4934	0.9720	-0.5520	0.4637	0.2339	0.3401	0.3491	0.3300
Apero, chilled (cheese, salami, humus, olives etc.)	E	-0.1320	0.3025	0.6627	-0.4445	0.2893	0.1244	-0.0068	0.2338	0.9769
Frozen vegetables and soups	A	0.6131	0.2124	0.0039	0.6354	0.2126	0.0028	0.5572	0.1747	0.0014
Frozen vegetables and soups	B	-0.7845	0.1724	<.0001	-0.7955	0.1725	<.0001	-0.7033	0.1406	<.0001
Frozen vegetables and soups	C	0.1549	0.1054	0.1417	0.1474	0.1055	0.1623	0.1291	0.1006	0.1993
Frozen vegetables and soups	E	0.0747	0.0601	0.2143	0.0706	0.0599	0.2392	0.1014	0.0554	0.0673
Canned vegetables	A	0.0068	0.0705	0.9227	0.0214	0.0704	0.7613	-0.0585	0.0603	0.3319
Canned vegetables	B	-0.0581	0.0544	0.2856	-0.0701	0.0543	0.1965	-0.0074	0.0484	0.8781
Canned vegetables	C	0.0511	0.0441	0.2460	0.0488	0.0441	0.2684	0.0655	0.0386	0.0897
Ready-to-use fresh vegetables, potatoes	A	0.4015	0.0845	<.0001	0.3975	0.0846	<.0001	0.3962	0.0744	<.0001
Ready-to-use fresh vegetables, potatoes	B	-0.1573	0.0571	0.0059	-0.1759	0.0569	0.0020	-0.1752	0.0443	<.0001
Ready-to-use fresh vegetables, potatoes	C	-0.1448	0.0423	0.0006	-0.1230	0.0419	0.0033	-0.1409	0.0383	0.0002
Ready-to-use fresh vegetables, potatoes	D	-0.0956	0.0274	0.0005	-0.0894	0.0273	0.0011	-0.0656	0.0263	0.0126
Soups, canned	A	0.2107	0.1181	0.0745	0.1597	0.1173	0.1735	0.2037	0.0995	0.0407
Soups, canned	B	-0.5133	0.3305	0.1205	-0.4689	0.3307	0.1563	-0.3815	0.2743	0.1643
Soups, canned	C	0.3264	0.3224	0.3114	0.3842	0.3219	0.2327	0.2176	0.2738	0.4268
Soups, canned	D	-0.2112	0.0831	0.0110	-0.2443	0.0825	0.0031	-0.2187	0.0757	0.0039
Soups, canned	E	0.1663	0.0776	0.0321	0.1538	0.0776	0.0475	0.1551	0.0732	0.0341
Fresh fruit	A	0.0391	0.0057	<.0001	0.0410	0.0056	<.0001	0.03894	0.0052	<.0001
Fresh fruit	B	-0.0025	0.0054	0.6430	-0.0041	0.0054	0.4463	-0.00230	0.0041	0.5746
Fresh fruit	C	-0.0370	0.0071	<.0001	-0.0371	0.0071	<.0001	-0.0357	0.0068	<.0001
Canned fruit	A	0.2105	0.4202	0.6164	0.3451	0.4189	0.4100	-0.2449	0.2540	0.3350
Canned fruit	B	0.3954	0.2753	0.1510	0.5164	0.2732	0.0588	0.5992	0.2099	0.0043
Canned fruit	C	-0.6337	0.2891	0.0284	-0.8637	0.2810	0.0021	-0.4091	0.1734	0.0184
Canned fruit	D	0.0749	0.1142	0.5121	0.0622	0.1145	0.5871	0.2013	0.0951	0.0345
Dried fruit (including nuts)	A	-0.8127	0.2967	0.0062	-0.9576	0.2929	0.0011	-0.7125	0.2619	0.0065
Dried fruit (including nuts)	B	1,5572	0.3774	<.0001	1,6883	0.3751	<.0001	1,7205	0.3269	<.0001
Dried fruit (including nuts)	C	-1,1118	0.2584	<.0001	-1,0923	0.2587	<.0001	-1,2457	0.2255	<.0001
Dried fruit (including nuts)	D	0.4126	0.1423	0.0037	0.4152	0.1424	0.0036	0.2910	0.1280	0.0230
Dried fruit (including nuts)	E	-0.0783	0.0234	0.0008	-0.0730	0.0232	0.0017	-0.0907	0.0229	<.0001
Bread preparations (i.e. crackers, rusks)	A	0.0440	0.2506	0.8607	0.1100	0.2499	0.6599	-0.0801	0.1948	0.6809
Bread preparations (i.e. crackers, rusks)	B	0.0789	0.2032	0.6980	0.1195	0.2030	0.5560	0.0150	0.1837	0.9347
Bread preparations (i.e. crackers, rusks)	C	-0.7703	0.3248	0.0177	-0.6024	0.3212	0.0608	-0.8263	0.2745	0.0026
Bread preparations (i.e. crackers, rusks)	D	0.6310	0.2558	0.0137	0.5367	0.2541	0.0347	0.6705	0.2252	0.0029
Bread preparations (i.e. crackers, rusks)	E	0.0060	0.2438	0.9805	-0.1614	0.2386	0.4989	0.2314	0.1733	0.1817
Sweet Bread toppings (chocolate, jam, honey)	A	0.0053	0.0557	0.9240	0.0117	0.0558	0.8337	0.0164	0.0544	0.7625
Sweet Bread toppings (chocolate, jam, honey)	B	0.0669	0.0641	0.2967	0.0751	0.0642	0.2413	0.0893	0.0575	0.1209
Sweet Bread toppings (chocolate, jam, honey)	C	0.5599	0.1829	0.0022	0.6189	0.1823	0.0007	0.4750	0.1377	0.0006
Sweet Bread toppings (chocolate, jam, honey)	D	-0.1130	0.2210	0.6092	-0.1847	0.2198	0.4008	0.0375	0.1784	0.8334
Sweet Bread toppings (chocolate, jam, honey)	E	-0.5709	0.2070	0.0059	-0.5546	0.2072	0.0074	-0.6545	0.1680	<.0001
Spreadable salads (i.e. meat, crab, etc.)	B	-0.1509	0.1326	0.2553	-0.1200	0.1325	0.3651	-0.1773	0.1193	0.1374
Spreadable salads (i.e. meat, crab, etc.)	C	0.3859	0.1990	0.0525	0.5075	0.1958	0.0096	0.3759	0.1656	0.0233
Spreadable salads (i.e. meat, crab, etc.)	D	-0.2452	0.2384	0.3037	-0.4044	0.2344	0.0845	-0.2272	0.1885	0.2281
Breakfast cereals	A	-0.2902	0.2950	0.3253	-0.5973	0.2816	0.0340	-0.1351	0.1704	0.4280
Breakfast cereals	B	-0.3037	0.1370	0.0267	-0.3168	0.1371	0.0209	-0.3361	0.1166	0.0039
Breakfast cereals	C	0.0093	0.2967	0.9751	0.2545	0.2889	0.3784	-0.0299	0.2076	0.8856
Breakfast cereals	D	0.2795	0.2498	0.2634	0.3468	0.2493	0.1643	0.1850	0.1854	0.3184
Breakfast cereals	E	0.1854	0.1014	0.0676	0.1946	0.1014	0.0549	0.1951	0.0883	0.0271
Sweet biscuits	A	-0.0510	0.0114	<.0001	-0.0513	0.0113	<.0001	-0.0517	0.0111	<.0001
Sweet biscuits	B	-0.0310	0.0265	0.2422	-0.0365	0.0265	0.1686	-0.0480	0.0225	0.0327
Sweet biscuits	C	-0.1328	0.1615	0.4111	-0.1366	0.1616	0.3978	-0.0338	0.1417	0.8112
Sweet biscuits	D	-0.2728	0.1785	0.1264	-0.2840	0.1787	0.1120	-0.1601	0.1274	0.2091
Sweet biscuits	E	0.5433	0.1915	0.0046	0.6027	0.1912	0.0016	0.4927	0.1437	0.0006
Bread	A	-2,4176	0.4003	<.0001	-2,7543	0.3881	<.0001	-2,5210	0.3135	<.0001
Bread	B	3,1918	0.4632	<.0001	3,7976	0.4279	<.0001	3,0777	0.3335	<.0001
Bread	C	-0.3397	0.1581	0.0317	-0.4403	0.1556	0.0047	-0.3213	0.1089	0.0032
Bread	D	-0.4137	0.1798	0.0215	-0.5685	0.1741	0.0011	-0.2227	0.1256	0.0763
Bread	E	-0.0169	0.0241	0.4817	-0.0291	0.0238	0.2224	-0.0086	0.0175	0.6236
Viennese cakes and buns	C	0.1585	0.1064	0.1361	0.1656	0.1065	0.1200	0.1626	0.0944	0.0851
Viennese cakes and buns	D	-1,3587	0.3353	<.0001	-1,5010	0.3327	<.0001	-1,2374	0.2753	<.0001
Viennese cakes and buns	E	1,1424	0.3374	0.0007	1,2784	0.3351	0.0001	1,0215	0.2753	0.0002
Cakes and pastries	B	-0.5809	0.0577	<.0001	-0.5890	0.0577	<.0001	-0.5995	0.0553	<.0001
Cakes and pastries	C	-0.5268	0.4472	0.2388	-0.4750	0.4474	0.2885	-0.7537	0.2149	0.0005
Cakes and pastries	D	-0.6362	0.4215	0.1312	-0.5929	0.4218	0.1599	-0.3941	0.2388	0.0989
Cakes and pastries	E	1,4858	0.5046	0.0033	1,4032	0.5046	0.0054	1,4883	0.2866	<.0001
Confectionery	A	0.2202	0.0549	<.0001	0.2360	0.0547	<.0001	0.1971	0.0532	0.0002
Confectionery	B	0.0455	0.1912	0.8119	0.0438	0.1914	0.8189	0.0138	0.1651	0.9335
Confectionery	C	0.3513	0.1149	0.0022	0.3269	0.1148	0.0044	0.2997	0.1021	0.0034
Confectionery	D	-1,1470	0.3439	0.0009	-1,2357	0.3427	0.0003	-1,0050	0.2398	<.0001
Confectionery	E	0.5678	0.3554	0.1102	0.6599	0.3535	0.0620	0.5395	0.1985	0.0066
Chocolate and bars	C	0.0812	0.0702	0.2492	0.0948	0.07441	0.2044	0.0788	0.0655	0.2315
Chocolate and bars	D	0.2361	0.1227	0.0544	0.1638	0.1209	0.1756	0.1856	0.0946	0.0497
Chocolate and bars	E	-0.2341	0.1230	0.0570	-0.1603	0.1211	0.1858	-0.1808	0.0948	0.0565
Pasta, rice and starch products	A	-0.0194	0.1824	0.9152	-0.0353	0.1825	0.8465	-0.0643	0.1437	0.6543
Pasta, rice and starch products	B	0.1455	0.1317	0.2694	0.1935	0.1310	0.1397	0.1523	0.1052	0.1477
Pasta, rice and starch products	C	-0.1699	0.1138	0.1354	-0.2371	0.1121	0.0345	-0.1611	0.0910	0.0765
Pasta, rice and starch products	D	0.0484	0.0501	0.3342	0.0838	0.0490	0.0873	0.0763	0.0419	0.0690
Oil and vinegar	A	-0.1361	0.1702	0.4250	-0.1355	0.1714	0.4301	-0.1287	0.2641	0.6269
Oil and vinegar	B	-0.0051	0.0256	0.8436	0.0065	0.0255	0.8002	-0.0140	0.0234	0.5497
Oil and vinegar	C	0.4768	0.2506	0.0571	0.4768	0.2508	0.0573	0.5741	0.2162	0.0080
Oil and vinegar	D	-0.6273	0.2650	0.0179	-0.5995	0.2650	0.0237	-0.7291	0.2254	0.0012
Oil and vinegar	E	0.2540	0.0759	0.0008	0.2386	0.0759	0.0017	0.2534	0.0693	0.0003
Condiments and sauces	A	0.0207	0.1621	0.8985	0.1884	0.1547	0.2234	-0.0055	0.0866	0.9494
Condiments and sauces	B	0.0811	0.1191	0.4958	0.1404	0.1175	0.2321	0.1292	0.0800	0.1063
Condiments and sauces	C	-0.5212	0.2513	0.0381	-0.5925	0.2508	0.0182	-0.2467	0.1332	0.0641
Condiments and sauces	D	0.5276	0.3267	0.1064	0.4396	0.3260	0.1775	0.2666	0.1540	0.0835
Condiments and sauces	E	-0.1035	0.1564	0.5083	-0.1705	0.1548	0.2708	-0.1480	0.0910	0.1036
World food	A	-0.2429	0.1971	0.2179	-0.3628	0.1932	0.0604	-0.2685	0.1698	0.1139
World food	B	0.4007	0.2977	0.1784	0.6180	0.2905	0.0335	0.2968	0.2418	0.2198
World food	C	0.3526	0.2741	0.1983	0.4665	0.2718	0.0861	0.3949	0.2523	0.1176
World food	D	0.4252	0.2399	0.0763	0.2507	0.2341	0.2843	0.5186	0.2078	0.0126
World food	E	-0.9461	0.1788	<.0001	-0.9743	0.1788	<.0001	-0.9378	0.1753	<.0001
Sugar and sweeteners	B	0.7737	0.2377	0.0011	0.8664	0.2355	0.0002	0.8014	0.2163	0.0002
Sugar and sweeteners	C	0.3205	0.1854	0.0839	0.2861	0.1854	0.1230	0.2926	0.1730	0.0909
Sugar and sweeteners	D	-1,1171	0.2935	0.0001	-1,1397	0.2936	0.0001	-1,1233	0.2648	<.0001
Sugar and sweeteners	E	0.9878	10.049	0.3272	1,2651	1,0093	0.2120	1,0767	1,6221	0.5091
Flour and dessert preparations	A	0.1020	0.2939	0.7286	0.1035	0.2942	0.7251	0.3542	0.2480	0.1532
Flour and dessert preparations	B	-0.5819	0.2112	0.0059	-0.5538	0.2112	0.0088	-0.6586	0.1920	0.0006
Flour and dessert preparations	C	0.3938	0.2978	0.1861	0.4310	0.2978	0.1480	0.4783	0.2696	0.0760
Flour and dessert preparations	D	0.0003	0.3630	0.9994	0.0943	0.3619	0.7944	-0.2926	0.2819	0.2993
Flour and dessert preparations	E	0.0835	0.2850	0.7697	-0.0725	0.2820	0.7971	0.1127	0.2535	0.6565
Spreadable fats (i.e. butter, margarines)	C	-0.4370	0.1544	0.0047	-0.3221	0.1508	0.0328	-0.4964	0.0984	<.0001
Spreadable fats (i.e. butter, margarines)	D	0.0299	0.1953	0.8782	0.0532	0.1954	0.7853	0.0695	0.1381	0.6146
Spreadable fats (i.e. butter, margarines)	E	0.4046	0.2144	0.0592	0.2689	0.2109	0.2022	0.4268	0.1466	0.0036
Juices, fresh, chilled	B	-0.1710	0.1053	0.1045	-0.1871	0.1054	0.0758	-0.2219	0.0845	0.0086
Juices, fresh, chilled	C	0.8644	0.2612	0.0009	0.8439	0.2615	0.0013	0.9334	0.2108	<.0001
Juices, fresh, chilled	D	-0.2266	0.1842	0.2188	-0.2061	0.1843	0.2635	-0.1970	0.1624	0.2253
Juices, fresh, chilled	E	-0.4700	0.1275	0.0002	-0.4449	0.1274	0.0005	-0.5288	0.1147	<.0001
Fruit juices, ambient	A	-1,0311	0.1830	<.0001	-0.9741	0.1825	<.0001	-0.9697	0.1730	<.0001
Fruit juices, ambient	B	-0.0839	0.0953	0.3790	-0.0834	0.0954	0.3824	-0.0506	0.0853	0.5529
Fruit juices, ambient	C	0.9635	0.3448	0.0052	0.6901	0.3361	0.0401	0.8650	0.2617	0.0010
Fruit juices, ambient	D	0.3907	0.1230	0.0015	0.4690	0.1211	0.0001	0.3813	0.1131	0.0008
Fruit juices, ambient	E	-0.2603	0.2619	0.3202	-0.1130	0.2588	0.6624	-0.2492	0.2017	0.2168
Waters (including flavored)	A	0.0341	0.1552	0.8262	-0.0599	0.1528	0.6954	0.1141	0.0942	0.2258
Waters (including flavored)	B	-0.0968	0.1538	0.5294	-0.0122	0.1518	0.9360	-0.1787	0.0921	0.0524
Waters (including flavored)	D	-0.0461	0.0654	0.4809	-0.0412	0.0643	0.5220	-0.1247	0.0617	0.0435
Waters (including flavored)	E	-0.0533	0.0316	0.0915	-0.0488	0.0311	0.1172	-0.0527	0.0317	0.0967
Soft drinks (light, zero, regular)	A	-0.0207	0.0073	0.0047	-0.0210	0.0073	0.0041	-0.0159	0.0073	0.0293
Soft drinks (light, zero, regular)	B	0.0409	0.1650	0.8043	-0.0357	0.1638	0.8275	0.1402	0.1214	0.2482
Soft drinks (light, zero, regular)	C	-0.3746	0.0747	<.0001	-0.3982	0.0746	<.0001	-0.3861	0.0553	<.0001
Soft drinks (light, zero, regular)	D	0.4098	0.2075	0.0483	0.5017	0.2061	0.0150	0.2479	0.1348	0.0660
Soft drinks (light, zero, regular)	E	-0.0770	0.1845	0.6765	-0.0640	0.1847	0.7288	0.0072	0.1496	0.9617
Syrups	B	0.5046	0.2314	0.0293	0.5781	0.2308	0.0123	0.5246	0.2250	0.0198
Syrups	C	1,1152	0.5610	0.0469	12119	0.5604	0.0306	1,2054	0.4639	0.0094
Syrups	D	-0.4343	0.2222	0.0507	-0.4317	0.2224	0.0523	-0.3565	0.1993	0.0737
Syrups	E	-0.9215	0.5711	0.1067	-1,0027	0.5709	0.0791	-1,0742	0.4919	0.0290
Non –alcoholic drinks	B	0.2561	10.843	0.8133	1,1072	1,0572	0.2950	0.3784	0.9991	0.7049
Non –alcoholic drinks	C	-0.1647	0.4858	0.7345	0.1024	0.4773	0.8301	-0.1387	0.4642	0.7651
Non –alcoholic drinks	D	-0.4080	1,1724	0.7279	-0.8093	1,1673	0.4882	-0.5039	1,0561	0.6333
Non –alcoholic drinks	E	0.0227	1,0276	0.9824	0.2137	1,0280	0.8353	0.3086	0.9147	0.7358
Apero & Appetizers	A	-0.0427	0.7542	0.9549	0.2754	0.7508	0.7138	-1,2070	0.3342	0.0003
Apero & Appetizers	B	0.0276	0.6326	0.9652	-0.1827	0.6292	0.7715	0.0803	0.3325	0.8091
Apero & Appetizers	C	0.1253	0.4794	0.7938	0.4156	0.4720	0.3786	0.2019	0.3365	0.5485
Apero & Appetizers	D	0.0145	0.6484	0.9822	-0.1044	0.6486	0.8721	0.3698	0.5233	0.4798
Apero & Appetizers	E	-0.0098	0.7030	0.9888	-0.0917	0.7017	0.8960	0.7058	0.4353	0.1050
Meals to cook and heat (i.e. lasagna, goulash)	A	0.2759	0.3518	0.4329	0.4418	0.3472	0.2032	0.1752	0.1687	0.2990
Meals to cook and heat (i.e. lasagna, goulash)	B	-0.1286	0.3433	0.7080	-0.3628	0.3364	0.2809	-0.0452	0.1790	0.8008
Meals to cook and heat (i.e. lasagna, goulash)	C	0.0887	0.2685	0.7412	-0.0055	0.2674	0.9837	0.0517	0.1583	0.7440
Meals to cook and heat (i.e. lasagna, goulash)	D	-0.1961	0.3360	0.5595	-0.0537	0.3342	0.8723	-0.1528	0.1444	0.2899
Meals to cook and heat (i.e. lasagna, goulash)	E	-0.0197	0.0234	0.3991	-0.0160	0.0234	0.4943	-0.0333	0.0207	0.1083
Meals to go (i.e. wraps, salads, sandwiches)	A	1,0659	0.2883	0.0002	0.9520	0.2870	0.0009	1,1751	0.2337	<.0001
Meals to go (i.e. wraps, salads, sandwiches)	B	-0.9809	0.2219	<.0001	-0.9635	0.2221	<.0001	-1,0362	0.2088	<.0001
Meals to go (i.e. wraps, salads, sandwiches)	C	-0.1589	0.2537	0.5310	-0.0927	0.2533	0.7145	-0.1903	0.2260	0.3998
Meals to go (i.e. wraps, salads, sandwiches)	D	0.1319	0.0521	0.0114	0.1555	0.0518	0.0027	0.1560	0.0447	0.0005
Meals to go (i.e. wraps, salads, sandwiches)	E	-0.0474	0.0505	0.3479	0.0157	0.0472	0.7391	-0.0351	0.0376	0.3515
Meals concept & event	B	-0.5691	0.1671	0.0007	-0.5994	0.1672	0.0003	-0.6043	0.1592	0.0001
Meals concept & event	C	0.1592	0.3296	0.6291	0.3102	0.3264	0.3420	0.1154	0.3164	0.7154
Meals concept & event	D	1,1520	0.2988	0.0001	1,1094	0.2991	0.0002	1,2854	0.2842	<.0001
Meals to compose	A	-0.0130	0.2756	0.9624	-0.1617	0.2707	0.5501	0.2553	0.1686	0.1299
Meals to compose	B	1,7429	0.3933	<.0001	1,8010	0.3934	<.0001	2,0784	0.3562	<.0001
Meals to compose	C	-1,7789	0.4506	<.0001	-1,6561	0.4494	0.0002	-2,2211	0.3690	<.0001
Meals to compose	D	-0.3035	0.0894	0.0007	-0.2729	0.0882	0.0020	-0.2735	0.0866	0.0016

## Abbreviations

ESL: Electronic shelf labels
FOP: Front-of-pack
FVNL: Fruit vegetables nuts legumes
MTL: Multiple Traffic Lights
WHO: World Health Organization
